# A Comparison of Field-in-Field and Intensity Modulated Radiation Therapy in Delivering Hypofractionated Radiation Therapy for Prostate Cancer

**DOI:** 10.1016/j.adro.2023.101356

**Published:** 2023-08-15

**Authors:** Hawbir Ghafour, Jalil S. Ali, Ronak Taher Ali, Elhussien Sirelkhatim

**Affiliations:** a Zhianawa Cancer Center, Kurdistan, Iraq; b College of Medicine, Hawler Medical University, Erbil, Iraq; c Radiation and Isotopes Centre, Khartoum, Sudan

## Abstract

**Purpose:**

This study compares the dosimetric performance of the field-in-field (FIF) technique with intensity modulated radiation therapy (IMRT) for delivering hypofractionated radiation therapy to prostate patients with cancer. The FIF technique uses 6 beams, whereas IMRT uses 9 beams.

**Methods and Materials:**

This study was conducted on 15 patients with prostate cancer treated with step-and-shoot IMRT. The prescribed dose was 60 Gy in 20 fractions. The FIF plans contained 6 photon beams, and IMRT plans were designed using a 9-field step-and-shoot technique. Dose-volume histograms and dose distributions were evaluated to compare FIF and IMRT.

**Results:**

The results of the planning target volume indices analysis showed a significant difference in the maximum dose, dose to 2% of volume, and homogeneity index in favor of FIF and in the mean dose, dose to 98% of volume, and D95 in favor of IMRT. The results of the organs-at-risk analysis showed significant differences in the volume of the rectum and bladder receiving 60 Gy in favor of FIF and the volume of the rectum and femoral heads receiving 30 Gy, as well as the mean dose to the rectum, in favor of IMRT. IMRT had a higher median number of monitor units (MUs) and segments (886 MU, 64 segments) compared to FIF (434 MUs, 6 segments).

**Conclusions:**

The FIF technique and IMRT had comparable results in delivering hypofractionated radiation therapy for prostate cancer. The findings of this study may aid in decision-making for patients undergoing treatment.

## Introduction

Prostate cancer is the second most common cancer among men worldwide and the fourth most common cancer overall.^1^ Many patients with prostate cancer undergo radiation therapy as part of their treatment. Three-dimensional conformal radiation therapy (3DCRT) and intensity modulated radiation therapy (IMRT) are commonly used for delivering this therapy; IMRT has the potential to deliver a more conformal dose distribution,^2^^,^^3^ which can lead to improved tumor control and reduced adverse effects. However, the additional complexity of IMRT may also lead to increased delivery time and higher costs. To improve the dosimetric performance of 3DCRT, the field-in-field (FIF) technique is used by adding segments to the main fields, with the shape and weight adjusted through forward planning.^4^, ^5^, ^6^

In recent years, hypofractionated radiation therapy schedules, which involve delivering higher doses of radiation over a shorter period, have gained popularity as a treatment for prostate cancer.^7^, ^8^, ^9^ This approach has been shown to be as effective as conventional fractionated schedules,^10^^,^^11^ with the potential for reduced treatment time. The reduction of total dose in hypofractionation allows for improved geometric selectivity compared with traditional fractionation. This suggests that FIF dosimetric performance may be improved compared with conventional 3DCRT and IMRT. Studies have examined this hypothesis for different FIF and IMRT beam configurations.^11^, ^12^, ^13^, ^14^ In this study, we compared the dosimetric performance of 6-beam FIF with 9-beam IMRT.

## Methods and Materials

This study included 15 patients with prostate cancer in various stages (IIIA, IIIB, IIIC, IIC, or IIB) who were treated with step-and-shoot IMRT from January 2019 to March 2022. Table 1 provides the age, Gleason score, staging, and prostate-specific antigen for these patients.

**Table 1:** Patient characteristics

Patient no.	Age, y	Staging	PSA level, ng/mL	Gleason score
1	86	IIIA	34	8 (3 + 5)
2	71	IIIB	28	7 (3 + 4)
3	57	IIIA	43	7 (4 + 3)
4	67	IIIA	36	8 (4 + 4)
5	76	IIIC	>100	9 (4 + 5)
6	70	IIIC	31	9 (4 + 5)
7	74	IIIB	3	6 (3 + 3)
8	76	IIB	13	7 (3 + 4)
9	83	IIIC	42	9 (5 + 4)
10	74	IIIA	30	8 (4 + 4)
11	76	IIC	17	7 (4 + 3)
12	64	IIC	5	7 (4 + 3)
13	66	IIB	27	7 (3 + 4)
14	66	IIIA	30	7 (4 + 3)
15	64	IIIB	42	7 (3 + 4)

*Abbreviation:* PSA = Prostate-Specific Antigen.

All patients underwent computed tomography (CT) scans from the abdomen to the upper thigh with a slice thickness of 2.5 cm using the GE Optima CT simulator (GE Healthcare, Chalfont St. Giles, United Kingdom). The patients were positioned supine with a comfortably full bladder and empty rectum. They were instructed to drink approximately 500 to 700 mL of water 45 minutes before the CT scan and before each treatment session. The planning target volumes (PTVs) and organs at risk (OARs) were delineated by the same radiation oncologist. The clinical target volume included only the prostate for low-risk patients and the prostate in addition to seminal vesicles for other patients. A 7-mm margin was added to the clinical target volume in all directions to create the PTV, except at the posterior margin, where a 4-mm expansion was used. The OARs included in the study were the rectum, bladder, femoral heads, bowels, and penile bulb.

The prescribed dose for all patients was 60 Gy in 20 fractions, a hypofractionation regimen commonly used at our hospital. The clinical goals of this regimen were adopted from the modified preconception aims outlined by Pryor et al.^15^ The FIF plans were planned using the Xio 5 Treatment Planning System (Elekta AB, Stockholm, Sweden). Each of the FIF plans contained 6 photon beams with an energy of 10 MV and gantry angles of 22°, 90°, 168°, 194°, 270°, and 339°. For all 3DCRT plans, FIF was used as needed to achieve a uniform dose distribution and to satisfy clinical goals. The IMRT plans were designed using a 9-field step-and-shoot technique (gantry angles of 0°, 40°, 80°, 120°, 160°, 200°, 240°, 280°, and 320°) with a beam energy of 10 MV. The optimization was performed using the Monaco 5.11 Treatment Planning System (Elekta AB). The maximum number of segments per plan, minimum segment size, and minimum MUs per segment were restricted to 108, 4 cm^2^, and 5 MU, respectively.

The dose volume histograms and dose distributions of the plans were evaluated to compare FIF and IMRT. For the PTV, the following data were analyzed: maximum dose (D_max_); minimum dose; mean dose (D_mean_); dose to 98% of volume (D98); dose to 95% of volume (D95); dose to 2% of volume (D2); conformity index (CI), which was calculated as CI = V_TR_/TV (where V_TR_ is the volume of the reference dose and TV is the target volume)^16^; and homogeneity index (HI), which was calculated as HI = (D2 – D98)/D50 (where D50 is the dose to 50% of volume).^17^ For the OARs, the D_mean_ was reported for the rectum, bladder, left and right femoral heads, and penile bulb. The rectum was also analyzed for the volume receiving 60 Gy (V60), volume receiving 57 Gy, and volume receiving 30 Gy (V30). The bladder was analyzed for the volume receiving 60 Gy (V60), volume receiving 50 Gy, and volume receiving 40 Gy. The left and right femoral heads were analyzed for the V30.

The statistical package R, version 4.2.2 (R Foundation for Statistical Computing, Vienna, Austria), was used to apply a one-way analysis of variance test for all PTV and OAR parameters to evaluate the significance of the difference between the 2 techniques. A *P* value of <.05 was considered statistically significant. Additionally, the R package was used to assess and summarize the statistical characteristics of the number of MU and beam segments for FIF and IMRT plans.

## Results

**Figure 1:**
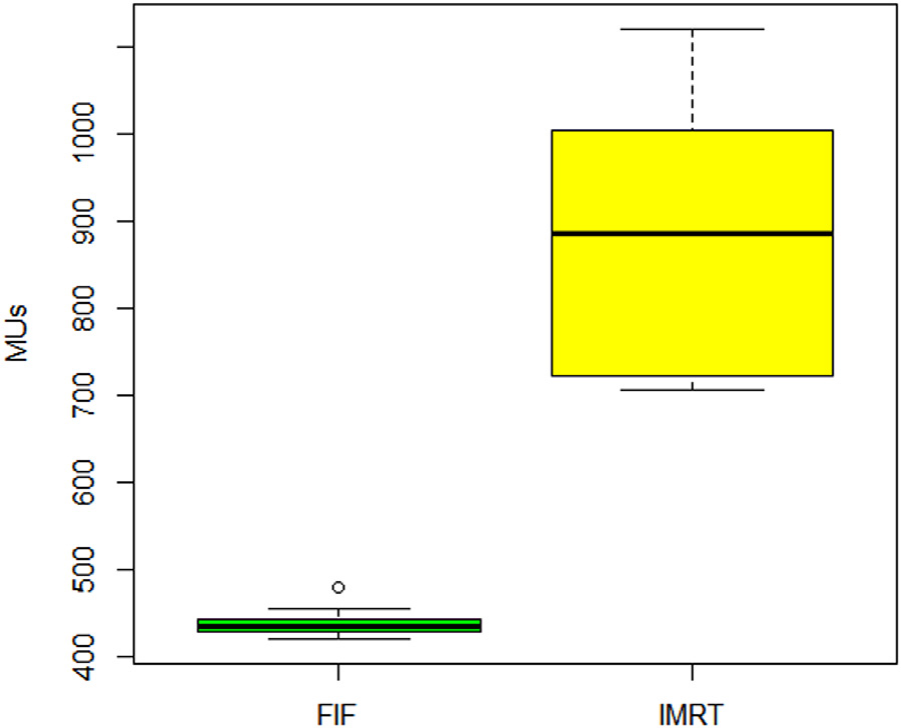
Box plots illustrating the distribution of MU for field-in-field (FIF) and intensity modulated radiation therapy (IMRT) plans.

**Figure 2:**
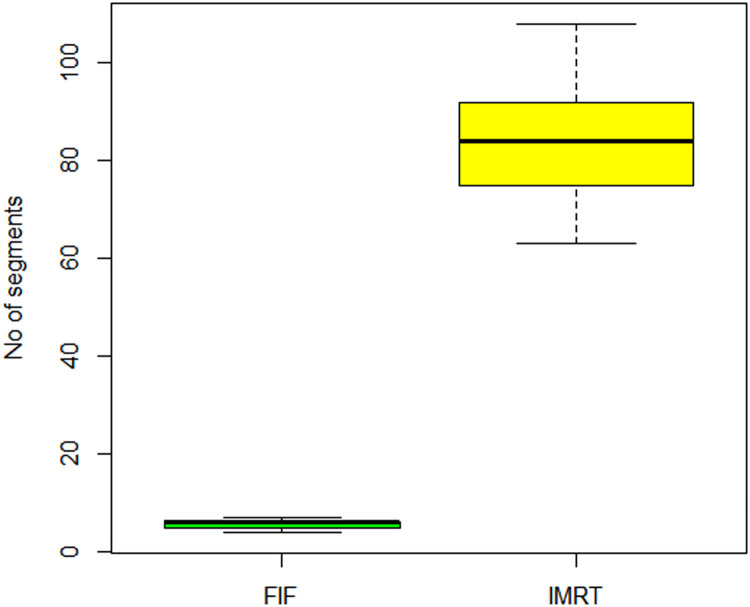
Box plots illustrating the distribution of number of beam segments for field-in-field (FIF) and intensity modulated radiation therapy (IMRT).

Results of this study showed in Table 2 that IMRT plans had higher D_max_, D_mean_, D98, D95, and D2 values compared with FIF plans (*P* < .05), with differences ranging from 0.73% to 5.85% (normalized to the prescribed dose, which was 60 Gy). However, there was no significant difference in minimum-dose values between the 2 techniques. The CI values for FIF plans were 1.06 ± 0.02 and for IMRT plans were 1.09 ± 0.03, with no significant difference between them. The FIF plans showed a more homogeneous dose distribution as evidenced by a lower HI value of 1.04 ± 0.02 compared with the IMRT value of 1.08 ± 0.03 (*P* < .05). Table 3 summarizes the dose-volume histogram analysis of the OAR and shows significant differences in V60 values for the rectum (*P* = .0191) and bladder (*P* = .0239) and significant differences in V30 values for the rectum (*P* = .00222) and the left (*P* = .00139) and right (*P* = .0392) femoral heads. Additionally, there was a significant difference in D_mean_ values for the rectum between the 2 techniques (*P* = .0345). Figure 1 demonstrates the difference in MU values for the FIF and IMRT plans, with FIF having a median of 434 and IQR of 12 and IMRT having a median of 886 and IQR of 281. Figure 2 shows the difference in the number of segments, with FIF having a median of 6 and IQR of 1 and IMRT having a median of 64 and IQR of 17.

**Table 2:** Summary of DVH analysis for the PTV

	3DCRT (mean **±** SD)	IMRT (mean **±** SD)	*P* value
D_min_ (Gy)	52.72 **±** 1.22	52.85 **±** 1.80	.959
D_max_ (Gy)	62.46 ± 1.19	65.97 ± 1.09	0
D_mean_ (Gy)	59.91 ± 0.57	61.81 ± 0.66	0
D98 (Gy)	56.77 ± 0.25	57.21 ± 0.50	.0345
D95 (Gy)	57.77 ± 0.22	58.34 ± 0.41	0
D2 (Gy)	61.99 ± 1.14	64.40 ± 1.36	0.0000616
CI	1.06 ± 0.3	1.06 ± 0.03	.849
HI	0.08 ± 0.02	0.12 ± 0.02	0

*Abbreviations:* 3DCRT = 3-dimensional conformal radiation therapy; CI = conformity index; D*x* = dose to *x*% of volume; D_max_ = maximum dose; D_mean_ = mean dose; D_min_ = minimum dose; DVH = dose volume histogram; HI = homogeneity index; IMRT = intensity modulated radiation therapy; PTV = planning target volume.

**Table 3:** Summary of DVH analysis for the OAR

OAR	**Dosimetric parameter**	FIF, mean **±** SD	IMRT, mean **±** SD	*P* value
Rectum	V60, %	0.29 ± 0.56	2.61 ± 2.99	.0191
	V57, %	9.72 ± 5.92	6.67 ± 4.11	.197
	V30, %	75.52 ± 11.06	59.92 ± 9.37	.00222
	D_mean_ (Gy)	37.45 ± 5.26	32.96 ± 3.77	.0345
Bladder	V60, %	2.37 ± 2.54	5.77 ± 3.94	.0239
	V50, %	21.77 ± 10.30	17.46 ± 6.87	.206
	V40, %	35.03 ± 15.29	28.99 ± 9.7	.211
	D_mean_, Gy	29.04 ± 8.94	27.57 ± 6.57	.479
Right femoral head	V30, %	0.54 ± 0.94	0.39 ± 0.68	.00139
	D_mean_, Gy	19.67 ± 3.32	14.83 ± 2.54	.578
Left femoral head	V30, %	0.39 ± 1.01	0.0 ± 0.0	.0392
	D_mean_, Gy	17.98 ± 4.47	15.26 ± 2.46	.194
Penile bulb	D_mean_, Gy	23.73 ± 8.82	22.35 ± 8.06	.884

*Abbreviations:* D_mean_ = mean dose; DVH = dose volume histogram; FIF = field-in-field; IMRT = intensity modulated radiation therapy; OAR = organ at risk; V*x* = volume receiving *x* Gy.

## Discussion

The results of this study indicate that both FIF and IMRT are effective methods for delivering hypofractionated radiation therapy to the prostate. Both techniques fulfilled the dose-prescription-aims treatment protocol.^15^ The statistically significant differences in the PTV coverage indices were in favor of FIF for D_max_, D2, and HI, which suggest better homogeneity compared with IMRT. On the other hand, IMRT was found to be superior in terms of D_mean_, D98, and D95. However, the wide margin of superiority in meeting the prescription aims minimizes the effects of these differences between the 2 techniques. Additionally, both techniques had a CI of less than 1.1 (1.06 ± 0.02 for FIF and 1.09 ± 0.03 for IMRT), which supports their comparability and meets 1 of the clinical goals of the treatment protocol.

The results also showed that the OAR indices favored FIF for V60 values in the rectum and bladder, whereas IMRT was favored in the other significant differences. The most pronounced differences in OAR dose distribution were evident in the rectum. The relatively large standard deviations for most OAR indices indicated the sensitivity of the 2 techniques to anatomic variations between patients and highlight the importance of following the instructions given to patients for getting an empty rectum and full bladder.

The clear differences in the number of MU and beam segments between FIF and IMRT were in favor of FIF, which suggests that this technique may be more practical and efficient, especially in busy departments or those with limited resources. The higher standard deviations of the number of MU and segments for IMRT suggest that this technique is more sensitive to anatomic variations between patients.

## Conclusions

The study found that both FIF and IMRT were effective for delivering hypofractionated radiation therapy to the prostate. Although FIF showed better results for D_max_, D2, and HI of the PTV and for the rectum and bladder V60, IMRT had better D_mean_, D98, and D95 of the PTV and some OAR parameters.

The anatomic variation between patients was found to affect the results, and following instructions to have an empty rectum and full bladder can reduce this variability.

In terms of the number of MU and beam segments, the results indicate that FIF has an advantage over IMRT, and this can be a significant factor in busy departments or those with limited resources. These results support the usability of FIF as a viable technique for delivering hypofractionated radiation therapy to the prostate.

## Disclosures

The authors declare that they have no known competing financial interests or personal relationships that could have appeared to influence the work reported in this paper.
